# Hepatitis E Virus Seroprevalence and Chronic Infections in Patients with HIV, Switzerland

**DOI:** 10.3201/eid1706.101067

**Published:** 2011-06

**Authors:** Alain Kenfak-Foguena, Franziska Schöni-Affolter, Philippe Bürgisser, Andrea Witteck, Katharine E.A. Darling, Helen Kovari, Laurent Kaiser, John-Marc Evison, Luigia Elzi, Vanina Gurtner De La Fuente, Josef Jost, Darius Moradpour, Florence Abravanel, Jacques Izopet, Matthias Cavassini, the Swiss HIV Cohort Study

**Affiliations:** Author affiliations: Centre Hospitalier Universitaire Vaudois and Lausanne University, Lausanne, Switzerland (A. Kenfak-Foguena, P. Bürgisser, K.E.A. Darling, D. Moradpour, M. Cavassini);; Data Center of the Swiss HIV Cohort Study, Lausanne (F. Schöni-Affolter);; Cantonal Hospital St. Gallen, St. Gallen, Switzerland (A. Witteck);; University of Zurich, Zurich, Switzerland (H. Kovari);; University Hospital Geneva, Geneva, Switzerland (L. Kaiser);; Inselspital Klinik und Poliklinik für Infektiologie, Bern, Switzerland (J.-M. Evison);; Universitätsspital Klinik für Infektiologie, Basel, Switzerland (L. Elzi);; Ospedale Civico Servizio Malattie Infettive, Lugano, Switzerland (V. Gurtner-De La Fuente);; Klinik Im Park, Zurich (J. Jost);; Centre Hospitalier Universitaire de Toulouse, Toulouse, France (F. Abravanel, J. Izopet)

**Keywords:** viruses, HIV, hepatitis, HEV, Switzerland, ALT elevation, alanine transaminase, liver enzymes, chronic infection, dispatch

## Abstract

We screened 735 HIV-infected patients in Switzerland with unexplained alanine aminotransferase elevation for hepatitis E virus (HEV) immunoglobulin G. Although HEV seroprevalence in this population is low (2.6%), HEV RNA can persist in patients with low CD4 cell counts. Findings suggest chronic HEV infection should be considered as a cause of persistent alanine aminotransferase elevation.

Unexplained liver enzyme elevation is frequently encountered in HIV-infected persons (*1*). Hepatitis E virus (HEV) can cause deranged liver function. Chronic infection with HEV has been reported in patients with organ transplants, treated malignancies, and HIV infection (*2*–*7*).

## The Study

The aim of this study was to investigate the prevalence and role of HEV infection among participants of the Swiss HIV Cohort Study enrolled up to December 2008 who had persistent elevated alanine aminotransferase (ALT) levels. Patients were eligible if they fulfilled the following criteria: 1) >18 years of age; 2) history of >2 consecutively elevated ALT values (>60 IU/L); and 3) negative for both hepatitis B antigen and hepatitis C virus antibody. Serum HEV immunoglobulin (Ig) G was detected by an enzyme immunoassay (EIA), the MP Diagnostics HEV ELISA Kit (MP Biomedicals, formerly Genelabs Diagnostics, Singapore). Positive samples were retested, and only those with a repeated positive result were considered positive. HEV RNA was determined in plasma by real-time PCR of a 189-bp product located in the open reading frame 2 region. Strains were sequenced and compared with reference HEV strains (GenBank) as described earlier (*8*).

For all patients meeting inclusion criteria, HEV serology was performed on the most recently stored blood sample. When available, for patients with positive serologic test results, HEV serology and blood HEV real-time PCR were performed on plasma samples stored 3 months before the first documented elevated ALT value. In these patients, HEV PCR was also performed on samples stored at the time of, and 3 months after, the first elevated ALT level.

To exclude occult infection, HEV PCR analysis was also performed on all available samples from IgG-negative patients with <150 CD4 cells/mm^3^ at the time of initial ALT elevation. To characterize more fully the patients with positive PCR, additional serologic testing was performed by using EIAgen HEV IgG and EIAgen HEV IgM kits (Adaltis Ingen, Paris, France).

Of 15,713 patients in the database, 2,000 patients had persistently elevated ALT values. Of these, 1,256 patients who were co-infected with hepatitis B virus or hepatitis C virus and 9 with missing data were excluded; 735 patients met the inclusion criteria (Table 1). IgG serologic tests were performed at a mean of 2.1 years after the first ALT elevation, and results were positive for 19 patients (2.6%). HEV-seropositive patients were more often female (p = 0.059) and of Asian origin (p = 0.007). In the univariate analysis, presence of HEV IgG was not associated with age, route of infection, lowest CD4 count, viral load, body mass index, or ALT values (Table 1). However, age- and gender-adjusted multivariate logistic regression analysis, with HEV IgG status as the outcome variable, yielded significant odds ratios for patients with low CD4 counts compared with patients with higher CD4 counts. Patients whose lowest CD4 counts were 100–350 cells/mm^3^ were 4.7 times more likely to be HEV positive compared with those with lowest CD4 counts <100 cells/mm^3^ (Table 2).

**Table 1:** Population characteristics in a study of prevalence and role of HEV infection among participants in the Swiss HIV Cohort Study, Switzerland, 2008*

Characteristic	No. (%) all participants, N = 735	No. (%) HEV negative, n = 716	No. (%) HEV positive, n = 19	p value
Sex				0.0587
M	618 (84.1)	605 (84.5)	13 (68.4)	
F	117 (15.9)	111 (15.5)	6 (31.6)	
Ethnic group				<0.0001
White	607 (82.6)	594 (83.0)	14 (73.7)	
Black	70 (9.5)	69 (9.6)	1 (5.3)	
Hispanic	26 (3.5)	25 (3.5)	1 (5.3)	
Asian	29 (3.9)	26 (3.6)	3 (15.8)	
Other	3 (0.4)	2 (0.28)	1 (0.14)	
Probable route of HIV infection				NS
Heterosexual	291 (39.6)	283 (39.5)	8 (42.1)	
MSM	411 (55.9)	400 (55.9)	11 (57.9)	
IDU	4 (0.6)	4 (0.6)	0	
Blood	5 (0.7)	5 (0.7)	0	
Unknown/other	24 (3.2)	24 (3.4)	0	
Current or past IDU				NS
Yes	14 (2.3)	14 (2.0)	0	
No	721 (99.7)	702 (98.0)	19 (100.0)	
Prison history				NS
Yes	45 (6.1)	44 (6.1)	1 (5.3)	
No	690 (93.9)	672 (93.9)	18 (94.7)	
Alcohol consumption				NS
Yes	261 (35.5)	256 (35.8)	5 (26.3)	
No	474 (64.5)	460 (64.2)	14 (73.7)	
BMI group, kg/m^2^				NS
<25	408 (55.5)	398 (55.6)	10 (52.6)	
25.1–30	231 (31.4)	225 (31.4)	6 (31.6)	
>30	96 (13.1)	93 (12.9)	3 (15.8)	
ALT peak value, IU/L				NS
<180	606 (82.4)	591 (82.5)	15 (78.95)	
>180	129 (17.6)	125 (17.5)	4 (21.05)	
Lowest CD4 count, cells/mm^3^				0.0685
<100	276 (37.6)	273 (38.1)	3 (15.8)	
100–350	288 (39.2)	276 (38.6)	12 (63.2)	
>350	171 (23.2)	167 (23.3)	4 (21.1)	
HAART history				NS
Yes	610 (83.0)	595 (75.4)	15 (78.9)	
No	125 (17.0)	121(16.9)	4 (21.6)	
Cancer occurrence				NS
Yes	25 (3.4)	24 (3.4)	1 (5.3)	
No	710 (96.6)	692 (94.6)	18 (94.7)	
Outcome				NS
Dead	29 (3.9)	28 (3.9)	1 (5.3)	
Alive	652 (88.7)	638 (89.1)	14 (73.7)	
Lost to follow-up	54 (7.4)	50 (7.0)	4 (21.1)	

*HEV, hepatitis E virus; MSM, men who have sex with men; IDU, intravenous drug use; BMI, body mass index; ALT, alanine aminotransferase; HAART, highly active antiretroviral therapy; NS, not significant.

**Table 2:** Logistic regression derived odds ratios/estimates for positive HEV serology in study of prevalence and role of HEV infection among participants in the Swiss HIV Cohort Study, Switzerland, 2008*

Variable	Odds ratio (95% CI)	p value
Male vs. female	0.207 (0.041–1.016)	0.0523
CD4 100–350 vs. CD4 <100 per mm^3^	4.683 (1.268–17.295)	0.0206
CD4 >350 vs. CD4 <100 per mm^3^	2.448 (0.522–11.468)	0.256
Other ethnicity vs. Asian ethnicity	0.295 (0.073–1.191)	0.0864
Alcohol history, no vs. yes	1.802 (0.582–5.581)	0.3071
Risk group, other vs. MSM	0.422 (0.100–1.774)	0.2392
Age at ALT elevation	1.017 (0.966–1.070)	0.5257
Duration of ALT elevation	1.001 (1.000–1.002)	0.0207

*HEV, hepatitis E virus; MSM, men who have sex with men; ALT, alanine aminotransferase.

In 16 of the 19 HEV seropositive patients, additional samples were available a median of 6.6 months (interquartile range [IQR] 4.5–6.0) before the first ALT elevation. Seroconversion from a prior negative to a positive result was found in 5 of these 16 patients (31.3%). There were no significant differences in ALT values (median, interval between and during high ALT periods), CD4 count, or HIV viral load of those who exhibited HEV seroconversion compared to those who did not.

**Figure 1:**
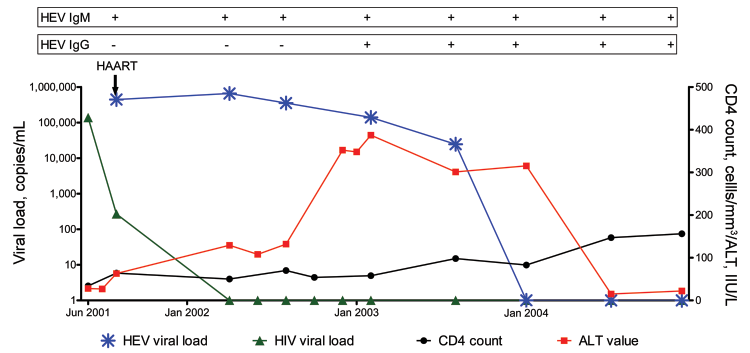
Longitudinal description of blood hepatitis E virus (HEV) serology, HEV RNA, alanine aminotransferase (ALT) levels, HIV RNA, and CD4 count in patient with chronic HEV infection, positive results by real-time PCR for HEV RNA, and seroconversion to immunoglobulin (Ig) G. HAART, highly active antiretroviral therapy.

Real-time PCR for HEV RNA was positive in 1 of the 19 HEV IgG–positive patients (genotype 3b), with presence of HEV RNA over a 24-month period, while HEV IgG remained negative for the first 12 months. This 46-year-old white man, who had sex with men (MSM) and received a diagnosis of HIV infection in June 2001, started highly active antiretroviral therapy (HAART) with a CD4 count of 34 cells/mm^3^ (5%) and a normal ALT level. ALT level increased 1 month later, but HAART was continued because the patient was asymptomatic. The patient refused a liver biopsy. Real-time PCR for HEV RNA was performed between August 2001 and December 2004. HEV PCR became negative for the virus when the CD4 count reached 83 cells/mm^3^ (17.5%), and ALT levels became normal 6 months later, when the CD4 count exceeded 100 cells/mm^3^. Figure 1 shows the course of ALT values, CD4 counts, and HIV and HEV plasma viral load.

**Figure 2:**
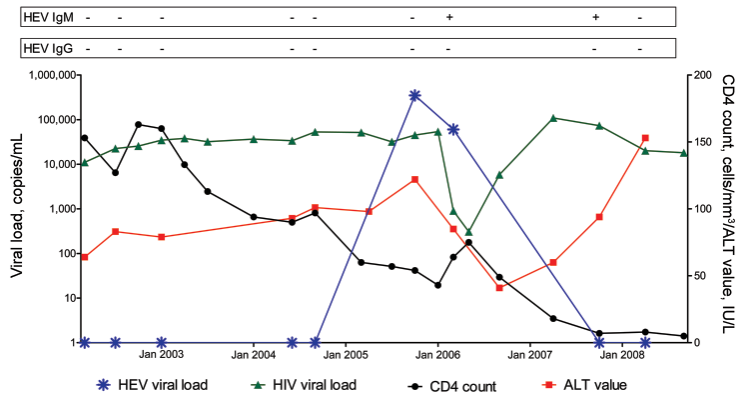
Longitudinal description of blood hepatitis E virus (HEV) serology, HEV RNA, alanine aminotransferase (ALT) levels, HIV RNA, and CD4 count in patient with positive real-time PCR results for HEV infection but without serologic seroconversion to immunoglobulin (Ig) G.

Given the prolonged viremia and late seroconversion (IgG) observed in patients with low CD4 counts, we performed real-time PCR for HEV RNA on 135 samples obtained from 54 HEV IgG–negative patients with low CD4 counts (<150 cells/mm^3^) at the time of initial ALT elevation. Real-time PCR for HEV was positive in 1 HEV IgG–negative patient over a 5-month period (Figure 2). This 59-year-old MSM started HAART in October 1996 with a CD4 count of 140 cells/mm^3^. CD4 counts remained <250 cells/mm^3^ over the subsequent 12 years. Real-time PCR for HEV RNA was performed between March 2002 and April 2008. HEV-positive samples (genotype 3c) were identified between October 2005 and March 2006. Additional serologic analysis revealed the presence of IgM over a 7-month period following first positive real-time PCR but absence of IgG seroconversion.

## Conclusions

This study of participants of the Swiss HIV Cohort Study shows a 2.6% (19/735) seroprevalence of HEV in HIV-infected patients without HBV or HCV infection with a history of persistently elevated ALT level. We did not find higher HEV prevalence in subgroups previously considered at higher risk of HEV infection such as MSM, injection drug users, and prisoners. The prevalence in Switzerland is lower than in other European countries and the United States (*9*). It is now well established that pigs and other animal species constitute reservoirs for HEV and that transmission in industrialized countries occurs mainly through contaminated meat. We hypothesize that strict regulation of animal imports in Switzerland (*10*) may reduce HEV prevalence among farm animals. Food preferences and possible regional factors may also contribute to the differences between countries and merit further study.

We observed anti-HEV seroconversion in 5 patients, including one with prolonged HEV RNA (>24 months). In this single patient, who had a very low CD4 count, seroconversion (IgG) was delayed until immune reconstitution occurred. As the CD4 count exceeded 100 cells/mm^3^, HEV RNA cleared, and ALT levels became normal (Figure 1). According to a literature search, 7 real-time PCR documented cases of HEV have been reported (*3*,*4*,*11*–*14*). Of these, 5 case-patients with CD4 counts >200 cells/mm^3^ sought treatment with acute infection and the virus cleared, whereas in 2 patients with <200 CD4 cells/mm^3^ persistent hepatitis developed (as we observed in our patient).

This study faced 2 common limitations regarding HEV diagnosis and comparability of seroprevalence: lack of an approved algorithm and variability of serologic tests in terms of sensitivity and specificity. According to a recent comparative study, the test used in our study may underestimate seroprevalence because of its inherent lower sensitivity (*15*). Moreover, the sensitivity of EIA tests among immunosuppressed patients is unknown and may be limited. We therefore screened all HEV IgG–negative patients (using HEV real-time PCR) with low CD4 counts at the time of unexplained, elevated ALT level and identified 1 patient who had 2 positive samples over a 5-month period. Unfortunately, later samples were not available to determine the precise duration of HEV viremia.

Taken together, our results suggest that serologic screening alone may be insufficient to diagnose HEV infection in HIV-infected patients with very low CD4 counts because seroconversion (IgG) may be delayed or not occur. When investigating unexplained, elevated ALT level in HIV-infected patients, we propose that HEV infection should be considered.
